# Changes in Serum C-Reactive Protein Following Nonsurgical Periodontal Therapy: A Systematic Review and Meta-Analysis

**DOI:** 10.3390/jcm15166251

**Published:** 2026-08-12

**Authors:** José María Montoya-Carralero, Rebeca Ávila-Villasmil, María Fernanda Romo-García, Alfonso Jornet-García, Arturo Sánchez-Pérez, María José Moya-Villaescusa

**Affiliations:** 1 Department of Periodontology, Faculty of Medicine, University of Murcia, 30008 Murcia, Spain; icuect@hotmail.com (J.M.M.-C.); alfonsofelipe.jornet@um.es (A.J.-G.); mjm.villaescusa@um.es (M.J.M.-V.); 2 Faculty of Medicine, University of Murcia, 30008 Murcia, Spain; rebeavilav@gmail.com; 3 Clinic for Dental Conservation, Periodontology and Preventive Dentistry, Faculty of Medicine, RWTH Aachen University, 52074 Aachen, Germany; fer.romog99@gmail.com

**Keywords:** periodontitis, periodontal therapy, C-reactive protein, systemic inflammation, meta-analysis, nonsurgical treatment

## Abstract

**Background:** Periodontitis is associated with elevated circulating C-reactive protein (CRP), but the extent to which CRP changes after nonsurgical periodontal therapy remains uncertain. This systematic review and meta-analysis examined within-participant changes in serum CRP and considered controlled evidence separately. **Methods:** PubMed/MEDLINE, the Cochrane Library, and SciELO were searched through August 2025. For uncontrolled pre-post studies reporting periodontitis-specific paired data, mean CRP reductions in mg/L were pooled using a random-effects model with restricted maximum likelihood and Hartung-Knapp confidence intervals. When the standard deviation (SD) of change was unavailable, it was calculated assuming a pre-post correlation of 0.50; correlations of 0.25 and 0.75 were examined in sensitivity analyses. Controlled trials were not pooled with uncontrolled studies. The protocol was retrospectively registered in the Open Science Framework before the quantitative analyses. **Results:** Five studies were included in the systematic review. Three uncontrolled studies comprising 119 participants contributed to the primary meta-analysis. The pooled within-participant reduction in CRP was 1.11 mg/L (95% CI: 0.21 to 2.01), with considerable heterogeneity (I^2^ = 80.8%). The result was stable across the assumed correlations. In the only trial comparing periodontal treatment with a control condition for which an effect could be calculated, the difference in mean changes was 2.95 mg/L (95% CI: −0.50 to 6.40) and was not statistically significant. **Conclusions:** CRP decreased after nonsurgical periodontal therapy in uncontrolled pre-post studies, but this finding does not establish a controlled treatment effect. Controlled evidence was limited and imprecise, and the clinical relevance of the biomarker change remains uncertain.

## 1. Introduction

Periodontitis is a chronic multifactorial inflammatory disease associated with dysbiotic plaque biofilms and characterized by the progressive destruction of tooth-supporting tissues. Beyond its local effects, increasing evidence suggests that periodontitis may contribute to a systemic inflammatory burden through the dissemination of periodontal pathogens, bacterial products, and inflammatory mediators into the circulation [1,2].

The ulcerated epithelial lining of periodontal pockets is estimated to represent a substantial wound surface area, providing a potential route for the translocation of microorganisms and inflammatory molecules into the bloodstream [3]. Consequently, periodontitis is associated with elevated circulating levels of several inflammatory biomarkers, including C-reactive protein (CRP), interleukin-6 (IL-6), and tumor necrosis factor-alpha (TNF-α) [4,5,6].

Among these biomarkers, CRP is among the most extensively investigated indicators of systemic inflammation. In response to proinflammatory cytokines, particularly IL-6, CRP is synthesized primarily by the liver. CRP has been widely used as a biomarker of cardiovascular risk and low-grade systemic inflammation. Elevated CRP concentrations have been consistently reported in patients with periodontitis compared with those in periodontally healthy individuals, supporting the hypothesis that periodontal inflammation may contribute to systemic inflammation [7,8].

Several clinical studies have evaluated the effect of nonsurgical periodontal therapy on systemic inflammatory markers. Previous evidence suggests that periodontal treatment may reduce circulating inflammatory mediators and improve surrogate markers of endothelial function [7,8]. However, the magnitude and consistency of CRP reduction following periodontal therapy remain uncertain. Published studies differ considerably in study design, patient characteristics, systemic health status, periodontal treatment protocols, follow-up periods, and methods for CRP assessment, leading to heterogeneous findings.

Although previous investigations have explored the relationship between periodontal treatment and systemic inflammation, an updated quantitative synthesis focused specifically on changes in serum CRP levels following nonsurgical periodontal therapy remains warranted. Clarifying this association is clinically relevant given the recognized role of systemic inflammation in cardiovascular and other chronic inflammatory diseases.

Therefore, this systematic review and meta-analysis aimed to quantify within-participant changes in serum CRP following nonsurgical periodontal therapy and to evaluate controlled comparisons separately when sufficient data were available.

## 2. Materials and Methods

### 2.1. Study Design

This systematic review and meta-analysis was conducted in accordance with the Preferred Reporting Items for Systematic Reviews and Meta-Analyses (PRISMA 2020) statement and the methodological guidance of the Cochrane Collaboration. The protocol was retrospectively registered in the Open Science Framework (OSF; DOI: 10.17605/OSF.IO/CP8BD). At the time of registration, the literature search and study selection had been completed, but the quantitative analyses had not yet been conducted. The eligibility criteria and outcomes were documented before the initial meta-analysis. During peer review, the statistical approach was revised to address methodological concerns, and all analytical changes are reported transparently in the revised manuscript and Supplementary Materials.

### 2.2. Inclusion and Exclusion Criteria

Clinical studies (randomized and nonrandomized) conducted within the last 15 years and published in English and/or Spanish were eligible for inclusion. Selection criteria were defined according to the PICO framework (population, intervention, comparison, and outcome). Population (P): adults older than 18 years with a clinical diagnosis of periodontitis, including chronic or severe periodontitis.

Intervention (I): Nonsurgical periodontal treatment, including scaling and root planing, periodontal debridement, conventional nonsurgical periodontal therapy, and full-mouth periodontal debridement protocols. Comparison (C): Two types of comparisons were considered: pretreatment versus posttreatment within the same patient group, and comparisons between treated and control groups when the study design allowed. Outcome (O): The primary outcome was the change in serum CRP levels following periodontal treatment.

Studies were eligible for the systematic review when they reported quantitative serum CRP outcomes. Inclusion in the quantitative synthesis additionally required extractable group-level or paired summary data sufficient to calculate a reproducible effect estimate.

### 2.3. Exclusion Criteria

Studies conducted in animals, systematic reviews and meta-analyses, studies without quantitative serum CRP outcomes, articles published in languages other than English or Spanish, and studies published before 2010 were excluded. Studies meeting the review criteria but lacking extractable summary statistics were retained for narrative synthesis when appropriate.

### 2.4. Search Strategy

On 26 August 2025, a systematic search was conducted in PubMed/MEDLINE, the Cochrane Library, and SciELO using combinations of the following terms and Boolean operators: ((periodontal disease) OR (periodontitis) OR (periodontal therapy) OR (periodontal treatment)) AND ((C-reactive protein) OR (CRP) OR (inflammatory markers) OR (systemic inflammation)). In addition, the reference lists of selected articles were manually reviewed to identify other potentially relevant studies.

### 2.5. Study Selection Process

**Figure 1:**
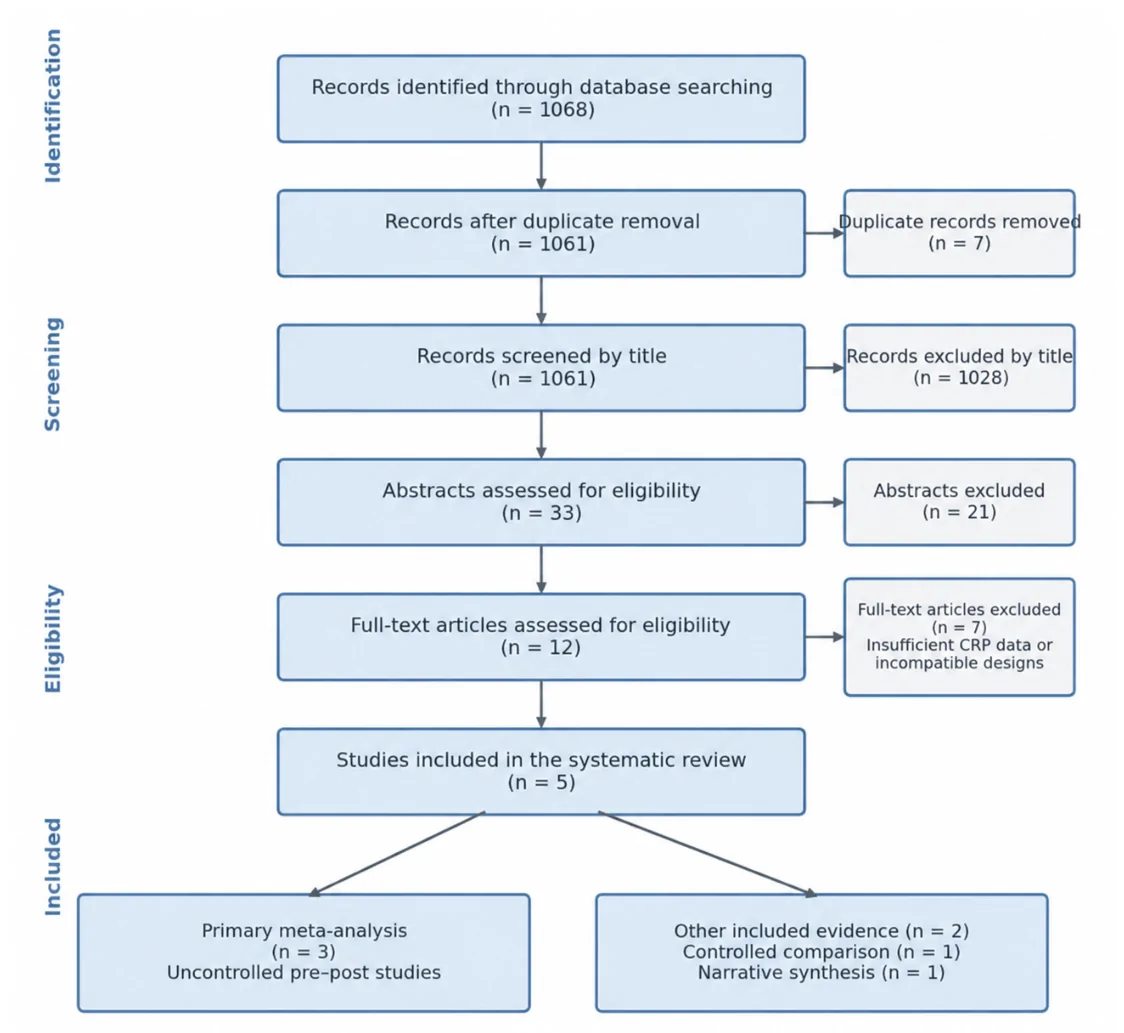
PRISMA flow diagram of the study selection process.

The study selection process consisted of two phases: an initial screening of titles and abstracts, followed by a full-text evaluation. The process was carried out by two independent reviewers, and discrepancies were resolved by consensus. The selection flow is presented in the PRISMA Statement diagram (Figure 1).

Initially, 1068 records were identified through database searching. After 7 duplicate records were removed, 1061 titles were screened, of which 1028 were excluded. Thirty-three abstracts were assessed for eligibility, and 21 were subsequently excluded. Twelve full-text articles were reviewed in detail, and seven were excluded because they did not meet the eligibility criteria, principally owing to insufficient extractable CRP data or incompatible study designs. Five studies were included in the systematic review. Three uncontrolled studies contributed to the primary meta-analysis, one randomized trial contributed a separate controlled comparison, and one active-comparator trial was summarized narratively. The study selection process is presented in Figure 1.

### 2.6. Data Extraction

Data were extracted into a standardized form recording author, year, country, study design, sample size, population, intervention and comparator, CRP assay, baseline and follow-up CRP values, SDs, published mean changes and SDs of change, and follow-up time. All values used in the revised quantitative synthesis were checked directly against the original reports. When periodontitis-specific subgroups were reported separately, subgroup means and SDs of change were combined using standard aggregation formulas. No values were estimated from graphical displays.

### 2.7. Assessment of the Risk of Bias

**Figure 2:**
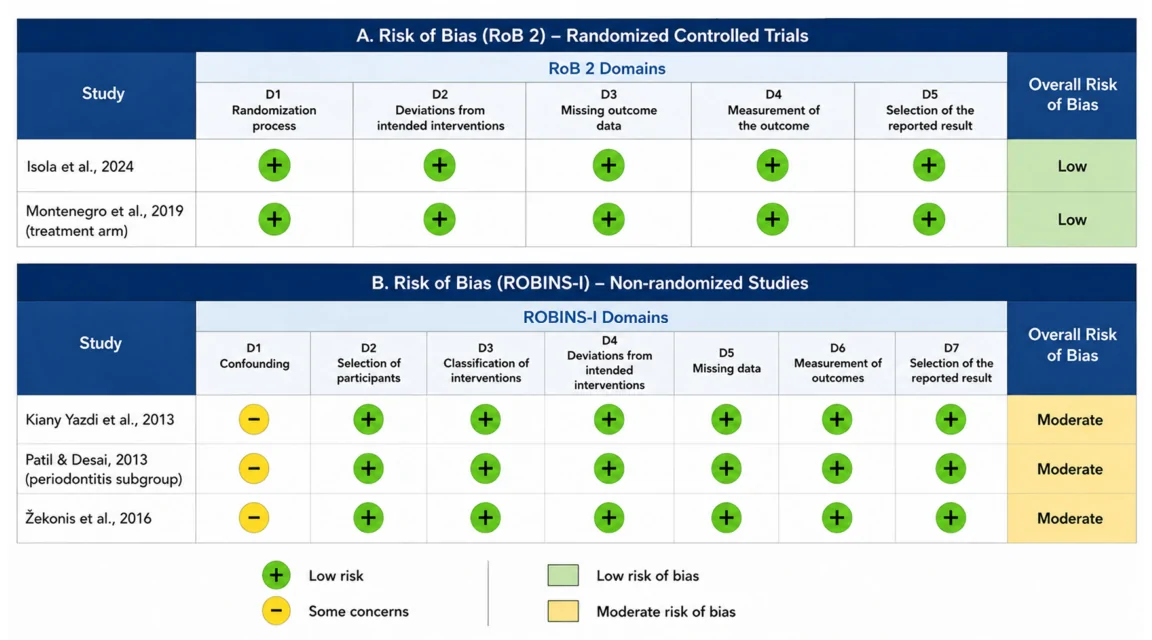
Risk-of-bias assessment of the included studies [9,10,11,12,13]. Randomized controlled trials were evaluated using the Cochrane Risk of Bias 2 (RoB2) tool, whereas nonrandomized studies were assessed using the ROBINS-I tool.

The risk of bias was assessed using the Cochrane Risk of Bias 2 (RoB 2) tool for randomized controlled trials and the ROBINS-I tool for nonrandomized studies. Each domain was rated as low risk, some concerns, or high risk of bias. Overall, the two randomized clinical trials were classified as low risk, whereas the three nonrandomized studies raised concerns primarily because of confounding and the absence of random allocation (Figure 2).

### 2.8. Statistical Analysis

The primary quantitative synthesis was restricted to uncontrolled pre-post studies that provided periodontitis-specific paired CRP data. Because all studies reported CRP on the same scale, the effect measure was the raw mean within-participant reduction from baseline to follow-up, expressed in mg/L; positive values indicate lower CRP at follow-up. Published mean changes and SDs of change were used when available.

For Kiany Yazdi et al., the mild, moderate, and severe periodontitis subgroups were combined, excluding participants with gingivitis. Patil and Desai reported the mean change and its SD directly. For Žekonis et al., the SD of change was calculated as SDchange = √(SDbaseline^2^ + SDfollow-up^2^ − 2r × SDbaseline × SDfollow-up), assuming a within-participant correlation of r = 0.50.

Mean changes were pooled using a random-effects model with between-study variance estimated by restricted maximum likelihood (REML). Hartung-Knapp adjustment with a t distribution was used for the pooled confidence interval because few studies were available. Heterogeneity was assessed using Cochran’s Q, τ^2^, and I^2^.

Statistical analyses were performed in Python 3.12 using SciPy 1.17.0. The verified study-level data, calculation details, and sensitivity analyses are provided in Supplementary Materials.

During revision, ChatGPT (GPT-5.6 Sol; OpenAI, San Francisco, CA, USA) was used to assist with language editing and the drafting of Python code for the statistical and graphical workflows. It was not used for study selection, data extraction, definition of the estimands, or generation or modification of source data. All code was executed by the authors, and all inputs, formulas, and numerical outputs were independently verified against the original reports and Supplementary Materials.

Controlled trials were evaluated separately because a difference in mean changes and an uncontrolled pre-post change estimate represent different estimands. For Montenegro et al., the difference between the mean change in the treatment group and that in the control group was calculated; SDs of change were estimated using r = 0.50. Isola et al. was retained in the narrative synthesis because it compared two active periodontal treatments and exact group-level CRP summary statistics required for a reproducible effect estimate were unavailable in the main report. No overall estimate combining controlled and uncontrolled designs was calculated.

### 2.9. Sensitivity Analysis

Sensitivity analyses repeated the calculations using pre-post correlations of r = 0.25 and r = 0.75 where change-score variability was not reported. Because only three studies were pooled, leave-one-out analyses, funnel plots, statistical tests for funnel-plot asymmetry, and trim-and-fill procedures were not performed.

## 3. Results

### 3.1. Study Selection Results

After applying the predefined inclusion and exclusion criteria, five studies were included in the systematic review. Three uncontrolled pre-post studies provided periodontitis-specific paired data suitable for the primary meta-analysis. The search identified 1068 records; after seven duplicates were removed, 1061 records were screened, 33 abstracts and 12 full texts were assessed, and seven full-text articles were excluded because of insufficient extractable CRP data or incompatible designs.

Kiany Yazdi et al. [9] (periodontitis subgroups; n = 65), Patil and Desai [10] (periodontitis group; n = 20), and Žekonis et al. [11] (n = 34) contributed to the pooled pre-post analysis, for a total of 119 participants. Montenegro et al. [12] contributed a separate controlled comparison involving 39 treated and 43 control participants. Isola et al. [13] included 21 participants in each active-treatment group and was summarized narratively because it compared two active treatments and the exact summary statistics required for recalculation were not reported in the main article.

### 3.2. Study Characteristics

The characteristics of the included studies are summarized in Table 1.

The studies differed in design, population, intervention, and follow-up. Kiany Yazdi et al. included patients receiving maintenance hemodialysis, Montenegro et al. included patients with stable coronary artery disease, and the remaining studies enrolled systemically healthy participants. Follow-up ranged from 8 weeks to 12 months. These differences were not treated as exchangeable through the random-effects model; controlled and uncontrolled evidence was therefore analyzed separately.

### 3.3. Individual Pre-Post Changes

Study-level mean reductions and 95% confidence intervals are presented in Table 2.

The mean reduction was 3.65 mg/L (95% CI: 1.27 to 6.03) in Kiany Yazdi et al., 1.21 mg/L (95% CI: 1.06 to 1.35) in Patil and Desai, and 0.96 mg/L (95% CI: 0.82 to 1.10) in Žekonis et al. The wide interval in Kiany Yazdi et al. reflects substantial variability among hemodialysis patients.

### 3.4. Heterogeneity

Considerable heterogeneity remained among the three pre-post studies (Q = 10.41, df = 2, *p* = 0.005; τ^2^ = 0.029; I^2^ = 80.8%).

### 3.5. Pooled Pre-Post Change

The three uncontrolled studies were pooled using the prespecified REML random-effects model with Hartung-Knapp adjustment.

The pooled within-participant reduction in serum CRP was 1.11 mg/L (95% CI: 0.21 to 2.01). This estimate describes change from baseline among treated participants and is not a controlled treatment effect.

### 3.6. Sensitivity Analyses

The pooled reduction was stable across correlations of 0.25, 0.50, and 0.75 (1.11, 1.11, and 1.11 mg/L, respectively), with overlapping confidence intervals. The controlled estimate from Montenegro et al. remained 2.95 mg/L, but its confidence interval narrowed as the assumed correlation increased and excluded zero only at r = 0.75. This dependence reinforces the uncertainty of the controlled estimate (Table 3).

### 3.7. Controlled Evidence

In Montenegro et al., CRP decreased by 2.49 mg/L in the treatment group and increased by 0.46 mg/L in the control group. With r = 0.50, the difference in mean changes was 2.95 mg/L (95% CI: −0.50 to 6.40) and was not statistically significant. Isola et al. reported a greater adjusted reduction with MINST than with quadrant-wise instrumentation, but exact summary statistics required for an independent effect calculation were unavailable in the main report; the study was therefore summarized narratively.

### 3.8. Forest Plot

**Figure 3:**
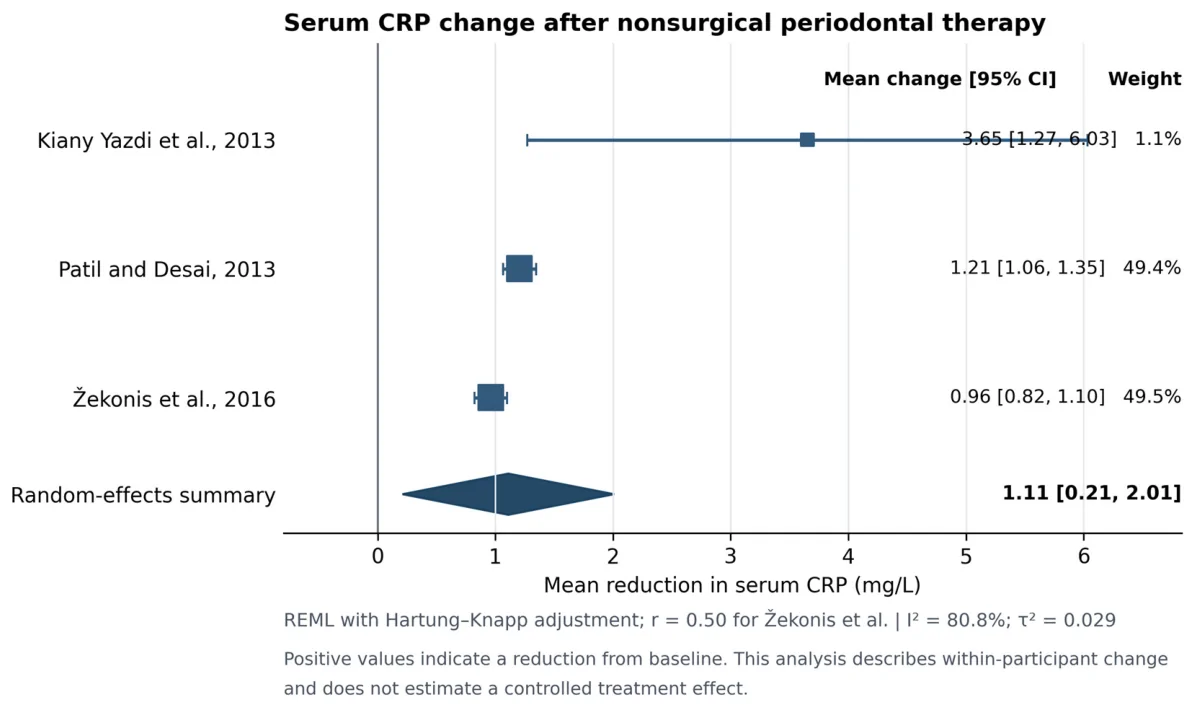
Forest plot of within-participant changes in serum C-reactive protein after nonsurgical periodontal therapy [9,10,11]. Positive values indicate lower CRP at follow-up. The pooled estimate was calculated using REML with Hartung-Knapp adjustment. This analysis does not estimate a controlled treatment effect.

The revised forest plot presents only the three uncontrolled pre-post studies and the pooled within-participant mean reduction (Figure 3).

### 3.9. Certainty of Evidence

The certainty of evidence for the pooled pre-post change was judged to be very low because the estimate was based on uncontrolled studies with considerable heterogeneity. The controlled evidence from Montenegro et al. was judged to be of moderate certainty but seriously imprecise because the confidence interval included both no effect and a potentially important reduction (Table 4).

## 4. Discussion

After complete re-extraction and recalculation, the primary synthesis found a pooled within-participant CRP reduction of 1.11 mg/L after nonsurgical periodontal therapy. However, the primary estimate was derived exclusively from three uncontrolled pre-post studies and therefore cannot distinguish a treatment effect from regression to the mean, temporal variation, changes in systemic health, or other concurrent influences.

The controlled evidence was less conclusive. In Montenegro et al., the difference in mean changes favored periodontal treatment but was imprecise and not statistically significant under the primary correlation assumption. Isola et al. compared two active treatment strategies and reported greater adjusted CRP reduction with MINST, but it did not provide a no-treatment comparison and could not be incorporated reproducibly into the quantitative analysis from the data reported in the main article.

### 4.1. Relationship Between Periodontitis and Systemic Inflammation

CRP is an acute-phase reactant primarily produced by the liver in response to proinflammatory cytokines, particularly IL-6. Chronic periodontal infection has been proposed to trigger the release of proinflammatory cytokines from periodontal tissues, which, in turn, stimulate hepatic CRP production [14]. In this context, periodontitis has been proposed as a potential contributor to low-grade systemic inflammation. Epidemiological and clinical studies have consistently shown elevated CRP levels in patients with periodontitis compared with those in periodontally healthy individuals [14,15]. Elevated CRP reflects a greater systemic inflammatory burden and has also been associated with cardiovascular risk; however, this relationship does not establish that periodontitis directly causes cardiovascular events.

### 4.2. Effects of Periodontal Treatment on CRP Levels

The pooled pre-post findings indicate that serum CRP was lower at follow-up among treated participants, which is consistent with previous reports of changes in systemic inflammatory markers after periodontal therapy. Nevertheless, the revised analysis separates this within-participant association from controlled treatment effects.

Other clinical studies have also reported changes in systemic inflammatory and cardiovascular biomarkers after periodontal therapy [16,17,18]. For example, Graziani et al. [18] described time-dependent changes in systemic inflammatory markers following periodontal interventions. Such findings support further investigation but do not demonstrate that CRP changes translate into improved systemic clinical outcomes.

### 4.3. Interpretation of Observed Heterogeneity

Considerable heterogeneity persisted in the revised pre-post analysis (I^2^ = 80.8%). Although lower than in the original calculation, this value indicates that the magnitude of CRP change was not uniform across studies.

The heterogeneity is clinically plausible. Kiany Yazdi et al. studied maintenance hemodialysis patients with high and highly variable baseline inflammation, whereas Patil and Desai and Žekonis et al. enrolled systemically healthier populations. The periodontal interventions, follow-up periods, and CRP assay methods also differed. The studies used nephelometry, immunoturbidimetry, or particle-enhanced turbidimetry, which may have introduced additional analytical variability. The random-effects model accounts for statistical dispersion but does not make these populations or designs clinically equivalent.

The sensitivity analysis showed that varying the assumed correlation had little effect on the pooled pre-post estimate because published change-score variability was available for Kiany Yazdi et al. and Patil and Desai; only Žekonis et al. required an assumed correlation. By contrast, the confidence interval for the single controlled estimate was sensitive to this assumption, highlighting its imprecision.

### 4.4. Clinical Implications

The observed reduction in serum CRP may indicate a change in systemic inflammatory status after periodontal treatment. However, CRP is a surrogate biomarker, and the available evidence does not establish that this reduction translates into fewer cardiovascular events or improved systemic clinical outcomes.

Nonsurgical periodontal therapy should therefore continue to be recommended primarily for the prevention and treatment of periodontal disease. Any potential systemic benefit should be regarded as an area for further investigation rather than as an established indication for periodontal treatment.

### 4.5. Strengths of the Study

The principal strength of the revised analysis is complete numerical traceability from the original reports to each study-level estimate. Periodontitis-specific data were used, published SDs of change were retained whenever available, assumptions were limited and tested in sensitivity analyses, and controlled and uncontrolled estimands were not pooled. The use of REML with Hartung-Knapp adjustment also provides a more conservative interval when only a few studies are available.

### 4.6. Limitations

This study has several limitations. Only three uncontrolled studies contributed to the primary meta-analysis, and their populations, interventions, and follow-up periods differed. The pooled estimate is susceptible to confounding, regression to the mean, and temporal changes unrelated to periodontal therapy. Considerable heterogeneity remained, and the small number of studies precluded meta-regression and any meaningful assessment of publication bias.

Controlled evidence was limited to one independently calculable comparison and was imprecise. The variance of the controlled difference required an assumed pre-post correlation, and the corresponding confidence interval was sensitive to that assumption. Isola et al. could not be recalculated from exact statistics available in the main report. Finally, CRP is a nonspecific surrogate biomarker, and no cardiovascular events or other systemic clinical outcomes were evaluated.

The protocol was retrospectively registered in the OSF after completion of the literature search and study selection, although registration preceded the quantitative analyses. This timing provides fewer transparency safeguards than prospective registration and does not fully exclude selective methodological decisions during the earlier stages of the review.

### 4.7. Clinical Relevance

The observed CRP reduction may reflect a change in systemic inflammatory status after periodontal treatment, but its clinical importance remains uncertain. No inference can be made regarding cardiovascular events or other systemic clinical outcomes.

## 5. Conclusions

Serum CRP decreased by an average of 1.11 mg/L in three uncontrolled pre-post studies following nonsurgical periodontal therapy. This finding describes within-participant change and should not be interpreted as evidence of a causal or uniform treatment effect. The available controlled comparison was imprecise and did not demonstrate a statistically significant difference under the primary correlation assumption. Further adequately powered randomized trials reporting complete change-score data and clinically relevant outcomes are needed.

## Figures and Tables

**Table 1:** Characteristics and quantitative role of the studies considered in the revised synthesis.

Study	Design	N	Population	CRP Assay	Baseline CRP (mg/L)	Follow-Up CRP (mg/L)	Follow-Up
Kiany Yazdi 2013 [9]	Uncontrolled pre-post	65	Hemodialysis patients with periodontitis	Nephelometry	NR for periodontitis-only sample	NR for periodontitis-only sample	8 weeks
Patil and Desai 2013 [10]	Prospective pre-post	20	Systemically healthy; periodontitis	Immunoturbidimetry	2.49 ± 0.47	1.30 ± 0.36	3 months
Žekonis 2016 [11]	Uncontrolled longitudinal	34	Systemically healthy; periodontitis	Particle-enhanced turbidimetry	2.48 ± 0.45	1.52 ± 0.36	1 year
Montenegro 2019 [12]	Randomized controlled	39/43	Stable coronary artery disease	Not reported	Treatment: 5.75 ± 9.77; control: 4.82 ± 7.01	Treatment: 3.26 ± 3.63; control: 5.28 ± 7.45	3 months
Isola 2024 [13]	Randomized active comparator	21/21	Stage III–IV periodontitis	Nephelometry	Adjusted estimates presented graphically	Adjusted estimates presented graphically	1 year

**Table 2:** Study-level within-participant changes in serum CRP included in the primary meta-analysis.

Study	N	Mean Reduction (mg/L)	SD Change	95% CI	Source of SD Change
Kiany Yazdi 2013 [9]	65	3.653	9.791	1.27 to 6.03	Reconstructed from published periodontitis subgroups
Patil and Desai 2013 [10]	20	1.205	0.320	1.06 to 1.35	Published directly
Žekonis 2016 [11]	34	0.960	0.412	0.82 to 1.10	Calculated with r = 0.50

**Table 3:** Sensitivity of the pooled pre-post estimate and controlled comparison to the assumed correlation.

Assumed r	Pooled Reduction (mg/L)	Pooled 95% CI	I^2^ (%)	Controlled Difference (mg/L)	Controlled 95% CI
0.25	1.11	0.22 to 2.01	78.2	2.95	−1.05 to 6.95
0.50	1.11	0.21 to 2.01	80.8	2.95	−0.50 to 6.40
0.75	1.11	0.20 to 2.01	83.8	2.95	0.16 to 5.74

Abbreviations: CI, confidence interval; CRP, C-reactive protein; I^2^, Higgins heterogeneity statistic.

**Table 4:** Certainty of evidence for uncontrolled pre-post change and controlled evidence.

Outcome	Studies (n)	Participants (n)	Certainty (GRADE)	Reasons
Within-participant CRP reduction	3	119	Very low	Uncontrolled designs; considerable inconsistency; limited sample
Controlled difference in CRP change	1	82	Moderate	Serious imprecision; single trial

Abbreviations: CRP, C-reactive protein; GRADE, Grading of Recommendations Assessment, Development and Evaluation.

## Data Availability

The verified extraction and calculation workbook supporting the revised analyses is provided as Supplementary Materials.

## Supplementary Materials

The following supporting information can be downloaded at: https://www.mdpi.com/article/10.3390/jcm15166251/s1.

## References

[1] Meyle J., Chapple I. (2015). Molecular aspects of the pathogenesis of periodontitis. Periodontol. 2000.

[2] Tonetti M.S., Jepsen S., Jin L., Otomo-Corgel J. (2017). Impact of the global burden of periodontal diseases on health, nutrition and wellbeing of mankind: A call for global action. J. Clin. Periodontol..

[3] Hujoel P.P., White B.A., García R.I., Listgarten M.A. (2001). The dentogingival epithelial surface area revisited. J. Periodontal Res..

[4] Herrera D., Molina A., Buhlin K., Klinge B. (2020). Periodontal diseases and association with atherosclerotic disease. Periodontol. 2000.

[5] Reyes L., Herrera D., Kozarov E., Roldán S., Progulske-Fox A. (2013). Periodontal bacterial invasion and infection: Contribution to atherosclerotic pathology. J. Periodontol..

[6] Schenkein H.A., Loos B.G. (2013). Inflammatory mechanisms linking periodontal diseases to cardiovascular diseases. J. Periodontol..

[7] Ling M.R., Chapple I.L., Matthews J.B. (2016). Neutrophil superoxide release and plasma C-reactive protein levels pre- and post-periodontal therapy. J. Clin. Periodontol..

[8] Orlandi M., Graziani F., D’Aiuto F. (2020). Periodontal therapy and cardiovascular risk. Periodontol. 2000.

[9] Kiany Yazdi F., Karimi N., Rasouli M., Roozbeh J. (2013). Effect of nonsurgical periodontal treatment on C-reactive protein levels in maintenance hemodialysis patients. Ren. Fail..

[10] Patil V.A., Desai M.H. (2013). Effect of periodontal therapy on serum C-reactive protein levels in patients with gingivitis and chronic periodontitis: A clinicobiochemical study. J. Contemp. Dent. Pract..

[11] Žekonis G., Žekonis J., Gleiznys A., Noreikienė V., Balnytė I., Šadzevičienė R., Narbutaitė J. (2016). Effect of supragingival irrigation with aerosolized 0.5% hydrogen peroxide on clinical periodontal parameters, markers of systemic inflammation, and morphology of gingival tissues in patients with periodontitis. Med. Sci. Monit..

[12] Montenegro M.M., Ribeiro I.W.J., Kampits C., Saffi M.A.L., Furtado M.V., Polanczyk C.A., Haas A.N., Rösing C.K. (2019). Randomized controlled trial of the effect of periodontal treatment on cardiovascular risk biomarkers in patients with stable coronary artery disease: Preliminary findings of 3 months. J. Clin. Periodontol..

[13] Isola G., Pesce P., Polizzi A., Lo Giudice A., Cicciù M., Scannapieco F.A. (2024). Effects of minimally invasive non-surgical therapy on C-reactive protein, lipoprotein-associated phospholipase A2, and clinical outcomes in periodontitis patients: A 1-year randomized, controlled clinical trial. J. Periodontol..

[14] Loos B.G. (2005). Systemic markers of inflammation in periodontitis. J. Periodontol..

[15] D’Aiuto F., Parkar M., Nibali L., Suvan J., Lessem J., Tonetti M.S. (2004). Periodontal infections cause changes in traditional and novel cardiovascular risk factors. J. Clin. Periodontol..

[16] Tonetti M.S., D’Aiuto F., Nibali L., Donald A., Storry C., Parkar M., Suvan J., Hingorani A., Vallance P., Deanfield J. (2007). Treatment of periodontitis and endothelial function. N. Engl. J. Med..

[17] D’Aiuto F., Nibali L., Parkar M., Suvan J., Tonetti M.S. (2005). Short-term effects of intensive periodontal therapy on serum inflammatory markers. J. Dent. Res..

[18] Graziani F., Cei S., Tonetti M., Paolantonio M., Serio R., Sammartino G., Gabriele M., D’Aiuto F. (2010). Systemic inflammation following non-surgical and surgical periodontal therapy. J. Clin. Periodontol..

